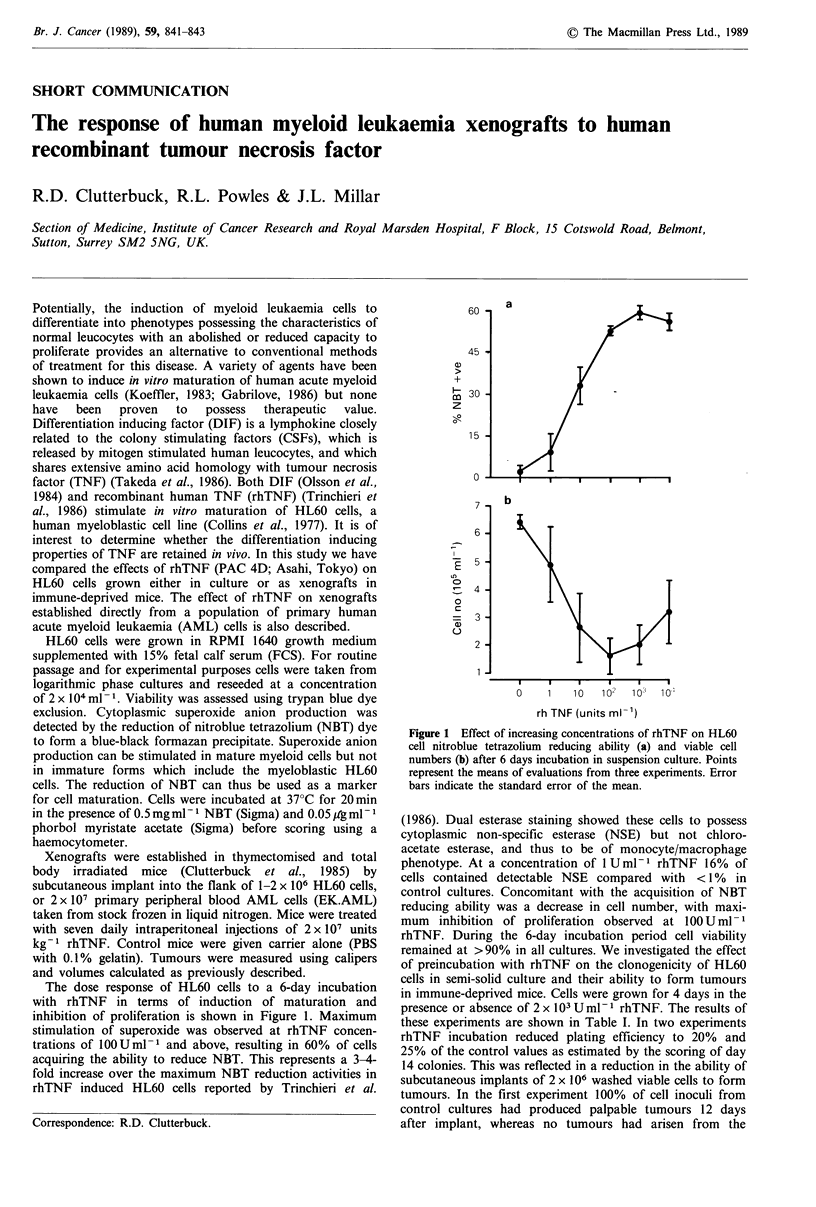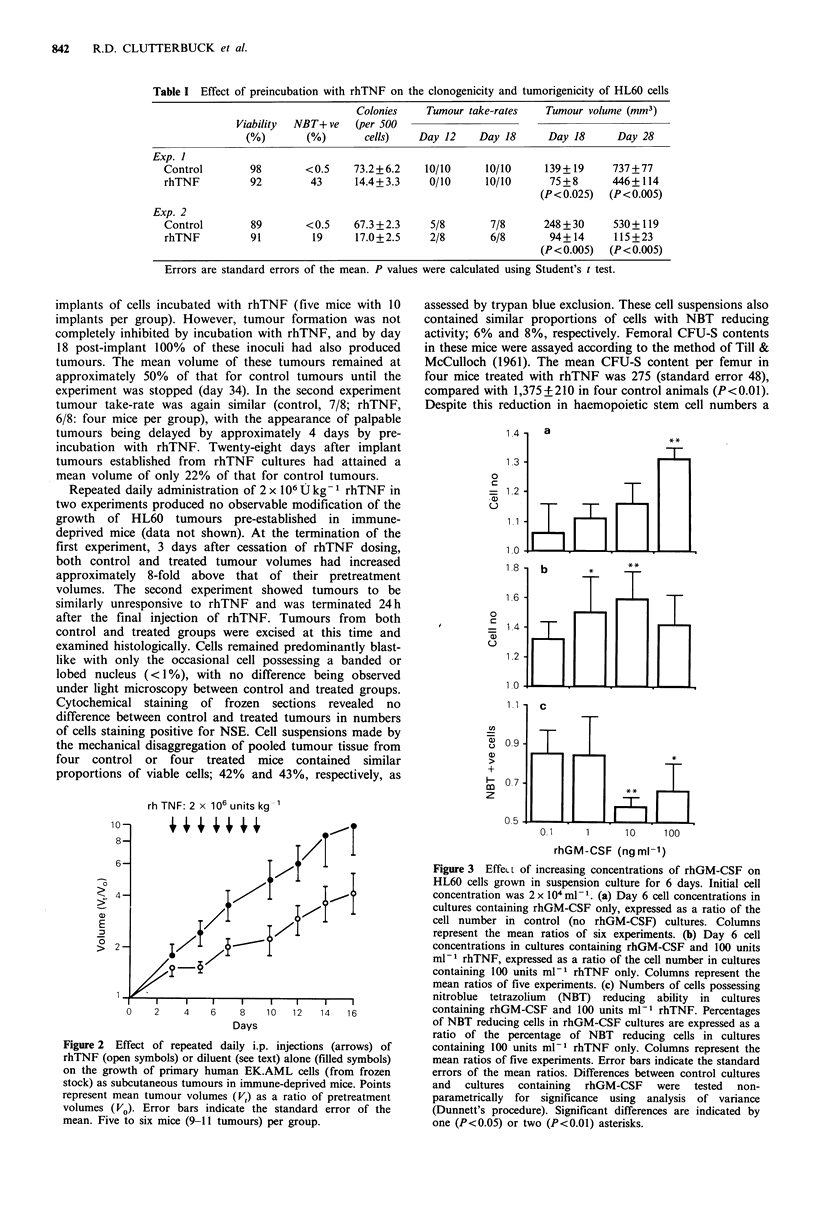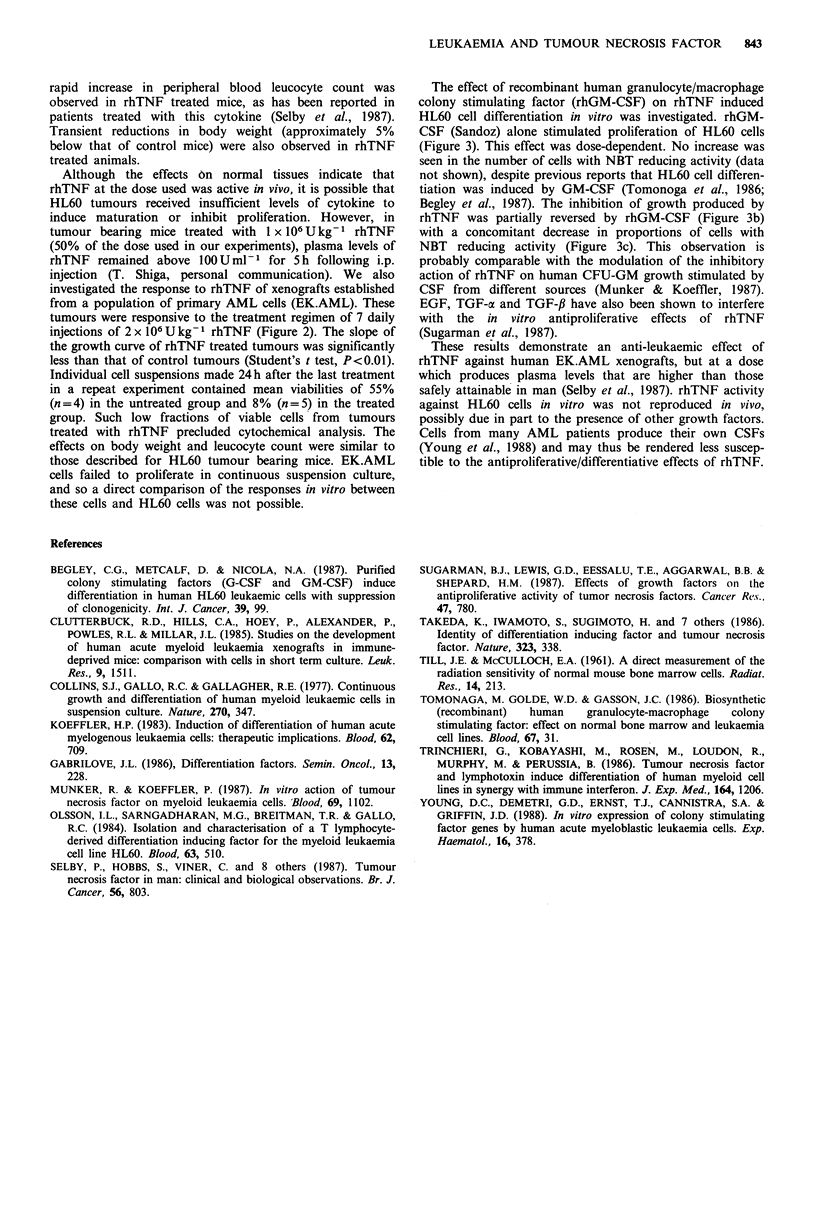# The response of human myeloid leukaemia xenografts to human recombinant tumour necrosis factor.

**DOI:** 10.1038/bjc.1989.179

**Published:** 1989-06

**Authors:** R. D. Clutterbuck, R. L. Powles, J. L. Millar

**Affiliations:** Section of Medicine, Institute of Cancer Research, Belmont, Sutton, Surrey, UK.


Br. J. Cancer (1989), 59, 841-843 © The Macmillan Press Ltd., 1989

SHORT COMMUNICATION

### The response of human myeloid leukaemia xenografts to human recombinant tumour necrosis factor

R.D. Clutterbuck, R.L. Powles & J.L. Millar

*Section of Medicine, Institute of Cancer Research and Royal Marsden Hospital, F Block, 15 Cotswold Road, Belmont, Sutton, Surrey SM2 5NG, UK.*

Potentially, the induction of myeloid leukaemia cells to differentiate into phenotypes possessing the characteristics of normal leucocytes with an abolished or reduced capacity to proliferate provides an alternative to conventional methods of treatment for this disease. A variety of agents have been shown to induce *in vitro* maturation of human acute myeloid leukaemia cells (Koeffler, 1983; Gabrilove, 1986) but none have been proven to possess therapeutic value. Differentiation inducing factor (DIF) is a lymphokine closely related to the colony stimulating factors (CSFs), which is released by mitogen stimulated human leucocytes, and which shares extensive amino acid homology with tumour necrosis factor (TNF) (Takeda *et al.*, 1986). Both DIF (Olsson *et al.*, 1984) and recombinant human TNF (rhTNF) (Trinchieri *et al.*, 1986) stimulate *in vitro* maturation of HL60 cells, a human myeloblastic cell line (Collins *et al.*, 1977). It is of interest to determine whether the differentiation inducing properties of TNF are retained *in vivo*. In this study we have compared the effects of rhTNF (PAC 4D; Asahi, Tokyo) on HL60 cells grown either in culture or as xenografts in immune-deprived mice. The effect of rhTNF on xenografts established directly from a population of primary human acute myeloid leukaemia (AML) cells is also described.

HL60 cells were grown in RPMI 1640 growth medium supplemented with 15% fetal calf serum (FCS). For routine passage and for experimental purposes cells were taken from logarithmic phase cultures and reseeded at a concentration of $2 \times 10^4$ ml$^{-1}$. Viability was assessed using trypan blue dye exclusion. Cytoplasmic superoxide anion production was detected by the reduction of nitroblue tetrazolium (NBT) dye to form a blue-black formazan precipitate. Superoxide anion production can be stimulated in mature myeloid cells but not in immature forms which include the myeloblastic HL60 cells. The reduction of NBT can thus be used as a marker for cell maturation. Cells were incubated at 37°C for 20 min in the presence of 0.5 mg ml$^{-1}$ NBT (Sigma) and 0.05 $\mu$g ml$^{-1}$ phorbol myristate acetate (Sigma) before scoring using a haemocytometer.

Xenografts were established in thymectomised and total body irradiated mice (Clutterbuck *et al.*, 1985) by subcutaneous implant into the flank of 1–2 $\times 10^6$ HL60 cells, or $2 \times 10^7$ primary peripheral blood AML cells (EK.AML) taken from stock frozen in liquid nitrogen. Mice were treated with seven daily intraperitoneal injections of $2 \times 10^7$ units kg$^{-1}$ rhTNF. Control mice were given carrier alone (PBS with 0.1% gelatin). Tumours were measured using calipers and volumes calculated as previously described.

The dose response of HL60 cells to a 6-day incubation with rhTNF in terms of induction of maturation and inhibition of proliferation is shown in Figure 1. Maximum stimulation of superoxide was observed at rhTNF concentrations of 100 U ml$^{-1}$ and above, resulting in 60% of cells acquiring the ability to reduce NBT. This represents a 3–4-fold increase over the maximum NBT reduction activities in rhTNF induced HL60 cells reported by Trinchieri *et al.*

**Figure 1** Effect of increasing concentrations of rhTNF on HL60 cell nitroblue tetrazolium reducing ability (**a**) and viable cell numbers (**b**) after 6 days incubation in suspension culture. Points represent the means of evaluations from three experiments. Error bars indicate the standard error of the mean.

(1986). Dual esterase staining showed these cells to possess cytoplasmic non-specific esterase (NSE) but not chloroacetate esterase, and thus to be of monocyte/macrophage phenotype. At a concentration of 1 U ml$^{-1}$ rhTNF 16% of cells contained detectable NSE compared with <1% in control cultures. Concomitant with the acquisition of NBT reducing ability was a decrease in cell number, with maximum inhibition of proliferation observed at 100 U ml$^{-1}$ rhTNF. During the 6-day incubation period cell viability remained at >90% in all cultures. We investigated the effect of preincubation with rhTNF on the clonogenicity of HL60 cells in semi-solid culture and their ability to form tumours in immune-deprived mice. Cells were grown for 4 days in the presence or absence of $2 \times 10^3$ U ml$^{-1}$ rhTNF. The results of these experiments are shown in Table I. In two experiments rhTNF incubation reduced plating efficiency to 20% and 25% of the control values as estimated by the scoring of day 14 colonies. This was reflected in a reduction in the ability of subcutaneous implants of $2 \times 10^6$ washed viable cells to form tumours. In the first experiment 100% of cell inoculi from control cultures had produced palpable tumours 12 days after implant, whereas no tumours had arisen from the

Correspondence: R.D. Clutterbuck.

**Table I** Effect of preincubation with rhTNF on the clonogenicity and tumorigenicity of HL60 cells

| | *Viability* (%) | *NBT+ve* (%) | *Colonies (per 500 cells)* | *Tumour take-rates* | | *Tumour volume (mm³)* | |
|---|---|---|---|---|---|---|---|
| | | | | *Day 12* | *Day 18* | *Day 18* | *Day 28* |
| *Exp. 1* | | | | | | | |
| Control | 98 | <0.5 | 73.2±6.2 | 10/10 | 10/10 | 139±19 | 737±77 |
| rhTNF | 92 | 43 | 14.4±3.3 | 0/10 | 10/10 | 75±8 ($P$<0.025) | 446±114 ($P$<0.005) |
| *Exp. 2* | | | | | | | |
| Control | 89 | <0.5 | 67.3±2.3 | 5/8 | 7/8 | 248±30 | 530±119 |
| rhTNF | 91 | 19 | 17.0±2.5 | 2/8 | 6/8 | 94±14 ($P$<0.005) | 115±23 ($P$<0.005) |

Errors are standard errors of the mean. $P$ values were calculated using Student's $t$ test.

implants of cells incubated with rhTNF (five mice with 10 implants per group). However, tumour formation was not completely inhibited by incubation with rhTNF, and by day 18 post-implant 100% of these inoculi had also produced tumours. The mean volume of these tumours remained at approximately 50% of that for control tumours until the experiment was stopped (day 34). In the second experiment tumour take-rate was again similar (control, 7/8; rhTNF, 6/8: four mice per group), with the appearance of palpable tumours being delayed by approximately 4 days by preincubation with rhTNF. Twenty-eight days after implant tumours established from rhTNF cultures had attained a mean volume of only 22% of that for control tumours.

Repeated daily administration of $2\times10^6$ U kg$^{-1}$ rhTNF in two experiments produced no observable modification of the growth of HL60 tumours pre-established in immune-deprived mice (data not shown). At the termination of the first experiment, 3 days after cessation of rhTNF dosing, both control and treated tumour volumes had increased approximately 8-fold above that of their pretreatment volumes. The second experiment showed tumours to be similarly unresponsive to rhTNF and was terminated 24 h after the final injection of rhTNF. Tumours from both control and treated groups were excised at this time and examined histologically. Cells remained predominantly blast-like with only the occasional cell possessing a banded or lobed nucleus (<1%), with no difference being observed under light microscopy between control and treated groups. Cytochemical staining of frozen sections revealed no difference between control and treated tumours in numbers of cells staining positive for NSE. Cell suspensions made by the mechanical disaggregation of pooled tumour tissue from four control or four treated mice contained similar proportions of viable cells; 42% and 43%, respectively, as assessed by trypan blue exclusion. These cell suspensions also contained similar proportions of cells with NBT reducing activity; 6% and 8%, respectively. Femoral CFU-S contents in these mice were assayed according to the method of Till & McCulloch (1961). The mean CFU-S content per femur in four mice treated with rhTNF was 275 (standard error 48), compared with 1,375±210 in four control animals ($P$<0.01). Despite this reduction in haemopoietic stem cell numbers a

**Figure 2** Effect of repeated daily i.p. injections (arrows) of rhTNF (open symbols) or diluent (see text) alone (filled symbols) on the growth of primary human EK.AML cells (from frozen stock) as subcutaneous tumours in immune-deprived mice. Points represent mean tumour volumes ($V_t$) as a ratio of pretreatment volumes ($V_0$). Error bars indicate the standard error of the mean. Five to six mice (9–11 tumours) per group.

**Figure 3** Effect of increasing concentrations of rhGM-CSF on HL60 cells grown in suspension culture for 6 days. Initial cell concentration was $2\times10^4$ ml$^{-1}$. (**a**) Day 6 cell concentrations in cultures containing rhGM-CSF only, expressed as a ratio of the cell number in control (no rhGM-CSF) cultures. Columns represent the mean ratios of six experiments. (**b**) Day 6 cell concentrations in cultures containing rhGM-CSF and 100 units ml$^{-1}$ rhTNF, expressed as a ratio of the cell number in cultures containing 100 units ml$^{-1}$ rhTNF only. Columns represent the mean ratios of five experiments. (**c**) Numbers of cells possessing nitroblue tetrazolium (NBT) reducing ability in cultures containing rhGM-CSF and 100 units ml$^{-1}$ rhTNF. Percentages of NBT reducing cells in rhGM-CSF cultures are expressed as a ratio of the percentage of NBT reducing cells in cultures containing 100 units ml$^{-1}$ rhTNF only. Columns represent the mean ratios of five experiments. Error bars indicate the standard errors of the mean ratios. Differences between control cultures and cultures containing rhGM-CSF were tested non-parametrically for significance using analysis of variance (Dunnett's procedure). Significant differences are indicated by one ($P$<0.05) or two ($P$<0.01) asterisks.

rapid increase in peripheral blood leucocyte count was observed in rhTNF treated mice, as has been reported in patients treated with this cytokine (Selby *et al.*, 1987). Transient reductions in body weight (approximately 5% below that of control mice) were also observed in rhTNF treated animals.

Although the effects on normal tissues indicate that rhTNF at the dose used was active *in vivo*, it is possible that HL60 tumours received insufficient levels of cytokine to induce maturation or inhibit proliferation. However, in tumour bearing mice treated with $1\times10^{6}\,\mathrm{U\,kg^{-1}}$ rhTNF (50% of the dose used in our experiments), plasma levels of rhTNF remained above $100\,\mathrm{U\,ml^{-1}}$ for 5h following i.p. injection (T. Shiga, personal communication). We also investigated the response to rhTNF of xenografts established from a population of primary AML cells (EK.AML). These tumours were responsive to the treatment regimen of 7 daily injections of $2\times10^{6}\,\mathrm{U\,kg^{-1}}$ rhTNF (Figure 2). The slope of the growth curve of rhTNF treated tumours was significantly less than that of control tumours (Student's $t$ test, $P<0.01$). Individual cell suspensions made 24h after the last treatment in a repeat experiment contained mean viabilities of 55% ($n=4$) in the untreated group and 8% ($n=5$) in the treated group. Such low fractions of viable cells from tumours treated with rhTNF precluded cytochemical analysis. The effects on body weight and leucocyte count were similar to those described for HL60 tumour bearing mice. EK.AML cells failed to proliferate in continuous suspension culture, and so a direct comparison of the responses *in vitro* between these cells and HL60 cells was not possible.

The effect of recombinant human granulocyte/macrophage colony stimulating factor (rhGM-CSF) on rhTNF induced HL60 cell differentiation *in vitro* was investigated. rhGM-CSF (Sandoz) alone stimulated proliferation of HL60 cells (Figure 3). This effect was dose-dependent. No increase was seen in the number of cells with NBT reducing activity (data not shown), despite previous reports that HL60 cell differentiation was induced by GM-CSF (Tomonoga *et al.*, 1986; Begley *et al.*, 1987). The inhibition of growth produced by rhTNF was partially reversed by rhGM-CSF (Figure 3b) with a concomitant decrease in proportions of cells with NBT reducing activity (Figure 3c). This observation is probably comparable with the modulation of the inhibitory action of rhTNF on human CFU-GM growth stimulated by CSF from different sources (Munker & Koeffler, 1987). EGF, TGF-$\alpha$ and TGF-$\beta$ have also been shown to interfere with the *in vitro* antiproliferative effects of rhTNF (Sugarman *et al.*, 1987).

These results demonstrate an anti-leukaemic effect of rhTNF against human EK.AML xenografts, but at a dose which produces plasma levels that are higher than those safely attainable in man (Selby *et al.*, 1987). rhTNF activity against HL60 cells *in vitro* was not reproduced *in vivo*, possibly due in part to the presence of other growth factors. Cells from many AML patients produce their own CSFs (Young *et al.*, 1988) and may thus be rendered less susceptible to the antiproliferative/differentiative effects of rhTNF.

### References

BEGLEY, C.G., METCALF, D. & NICOLA, N.A. (1987). Purified colony stimulating factors (G-CSF and GM-CSF) induce differentiation in human HL60 leukaemic cells with suppression of clonogenicity. *Int. J. Cancer*, **39**, 99.

CLUTTERBUCK, R.D., HILLS, C.A., HOEY, P., ALEXANDER, P., POWLES, R.L. & MILLAR, J.L. (1985). Studies on the development of human acute myeloid leukaemia xenografts in immune-deprived mice: comparison with cells in short term culture. *Leuk. Res.*, **9**, 1511.

COLLINS, S.J., GALLO, R.C. & GALLAGHER, R.E. (1977). Continuous growth and differentiation of human myeloid leukaemic cells in suspension culture. *Nature*, **270**, 347.

KOEFFLER, H.P. (1983). Induction of differentiation of human acute myelogenous leukaemia cells: therapeutic implications. *Blood*, **62**, 709.

GABRILOVE, J.L. (1986), Differentiation factors. *Semin. Oncol.*, **13**, 228.

MUNKER, R. & KOEFFLER, P. (1987). *In vitro* action of tumour necrosis factor on myeloid leukaemia cells. *Blood*, **69**, 1102.

OLSSON, I.L., SARNGADHARAN, M.G., BREITMAN, T.R. & GALLO, R.C. (1984). Isolation and characterisation of a T lymphocyte-derived differentiation inducing factor for the myeloid leukaemia cell line HL60. *Blood*, **63**, 510.

SELBY, P., HOBBS, S., VINER, C. and 8 others (1987). Tumour necrosis factor in man: clinical and biological observations. *Br. J. Cancer*, **56**, 803.

SUGARMAN, B.J., LEWIS, G.D., EESSALU, T.E., AGGARWAL, B.B. & SHEPARD, H.M. (1987). Effects of growth factors on the antiproliferative activity of tumor necrosis factors. *Cancer Res.*, **47**, 780.

TAKEDA, K., IWAMOTO, S., SUGIMOTO, H. and 7 others (1986). Identity of differentiation inducing factor and tumour necrosis factor. *Nature*, **323**, 338.

TILL, J.E. & McCULLOCH, E.A. (1961). A direct measurement of the radiation sensitivity of normal mouse bone marrow cells. *Radiat. Res.*, **14**, 213.

TOMONAGA, M. GOLDE, W.D. & GASSON, J.C. (1986). Biosynthetic (recombinant) human granulocyte-macrophage colony stimulating factor: effect on normal bone marrow and leukaemia cell lines. *Blood*, **67**, 31.

TRINCHIERI, G., KOBAYASHI, M., ROSEN, M., LOUDON, R., MURPHY, M. & PERUSSIA, B. (1986). Tumour necrosis factor and lymphotoxin induce differentiation of human myeloid cell lines in synergy with immune interferon. *J. Exp. Med.*, **164**, 1206.

YOUNG, D.C., DEMETRI, G.D., ERNST, T.J., CANNISTRA, S.A. & GRIFFIN, J.D. (1988). *In vitro* expression of colony stimulating factor genes by human acute myeloblastic leukaemia cells. *Exp. Haematol.*, **16**, 378.